# Disrupted methylation patterns at birth persist in early childhood: a prospective cohort analysis

**DOI:** 10.1186/s13148-022-01348-x

**Published:** 2022-10-15

**Authors:** Andrey V. Dolinko, Bryant M. Schultz, Jayashri Ghosh, Charikleia Kalliora, Monica Mainigi, Christos Coutifaris, Carmen Sapienza, Suneeta Senapati

**Affiliations:** 1 Department of Obstetrics and Gynecology, University of Pennsylvania, Philadelphia, PA USA; 2 Fels Cancer Institute for Personalized Medicine, Temple University, Philadelphia, PA USA

**Keywords:** DNA methylation, Epigenome, ART, IVF

## Abstract

**Background:**

Alterations in the epigenome are a risk factor in multiple disease states. We have demonstrated in the past that disruption of the epigenome during early pregnancy or periconception, as demonstrated by altered methylation, may be associated with both assisted reproductive technology and undesirable clinical outcomes at birth, such as low birth weight. We have previously defined this altered methylation, calculated based on statistical upper and lower limits of outlier CpGs compared to the population, as an ‘outlier methylation phenotype’ (OMP). Our aim in this study was to determine whether children thus identified as possessing an OMP at birth by DNA methylation in cord blood persist as outliers in early childhood based on salivary DNA methylation.

**Results:**

A total of 31 children were included in the analysis. Among 24 children for whom both cord blood DNA and salivary DNA were available, DNA methylation patterns, analyzed using the Illumina Infinium MethylationEPIC BeadChip (850 K), between cord blood at birth and saliva in childhood at age 6–12 years remain stable (*R*^2^ range 0.89–0.97). At birth, three out of 28 children demonstrated an OMP in multiple cord blood datasets and hierarchical clustering. Overall DNA methylation among all three OMP children identified as outliers at birth was remarkably stable (individual *R*^2^ 0.908, 0.92, 0.915), even when only outlier CpG sites were considered (*R*^2^ 0.694, 0.738, 0.828).

**Conclusions:**

DNA methylation signatures in cord blood remain stable over time as demonstrated by a strong correlation with epigenetic salivary signatures in childhood. Future work is planned to identify whether a clinical phenotype is associated with OMP and, if so, could undesirable clinical outcomes in childhood and adulthood be predicted at birth.

**Supplementary Information:**

The online version contains supplementary material available at 10.1186/s13148-022-01348-x.

## Background

More than eight million births have resulted from assisted reproductive technologies (ART) over the past four decades [1]. Although the vast majority of children who were conceived in vitro have no phenotype identified to date that demands clinical attention, an increased risk has been associated with rare imprinted gene disorders, such as Angelman and Beckwith–Wiedemann syndromes [2], as well as some adverse perinatal outcomes such as small for gestational age fetuses and low birth weight infants [3, 4]. Evidence now exists that these associations may be due to ‘environmental’ exposures during periconception, specifically the different ART procedures that are employed. Furthermore, these changes may affect early childhood development and possibly contribute to undesirable clinical outcomes in adulthood.

It is well established that the periconception period is associated with critical periods of epigenetic reprogramming, both in gametes and in embryos [5–9]. During these periods, the embryo may be particularly susceptible to environmentally induced epigenetic changes, including in response to controlled ovarian hyperstimulation, embryo culture, embryo biopsy for pre-implantation genetic testing, and embryo transfer, among others [9]. In parallel, studies have demonstrated that alterations in the epigenome are a risk factor in multiple disease states, including cancers, type 2 diabetes, schizophrenia, and autoimmune disease [10]. Taking these two points further, our group has demonstrated that disruption of the epigenome during early pregnancy or periconception, as identified by multiple and large DNA methylation differences, may be associated with both ART and undesirable clinical outcomes at birth, such as low birth weight [11].

We have previously established a prospective cohort of ART-conceived offspring and a non-ART control group. In that cohort, we identified that some offspring demonstrate an outlier methylation phenotype (OMP) at birth based on cord blood analysis [11]. The aim of this study was to determine whether children thus identified as possessing an OMP at birth by DNA methylation in cord blood persisted as outliers in early childhood based on salivary DNA methylation.

## Results

Twenty-eight cord blood samples and 28 salivary DNA samples from 31 children were available for methylation analysis and were included (see Materials and Methods for details). Of these, twenty-four had both cord blood and salivary DNA available. In this group, there were four pairs of twins (Families D, J, K, and O), and one set of twins (Family D) also had another sibling (Child E) included in the study. Demographics for the children included in this analysis are given in Table 1.

**Table 1:** Demographic characteristics of our cohort of children followed from birth to age 6–12 yr for DNA methylation studies

Demographic characteristic	
Sex*	
Male	13 (41.9%)
Female	18 (58.1%)
Maternal age (*y*)^	35.8 (4.4)
Paternal age (*y*)^	38.7 (4.9)
Mode of conception*	
Unassisted (non-ART)	2 (6.5%)
ART	29 (93.5%)
Fresh autologous embryo transfer	23 (74.2%)
Frozen autologous embryo transfer	3 (9.7%)
Donor egg embryo transfer	3 (9.7%)
Gestational age at birth^	38w6d (11d)
Birth weight (g)^	3352 (542)
Mode of delivery*	
Vaginal delivery	17 (54.8%)
Cesarean section	14 (45.2%)
Race/ethnicity*	
White non-Hispanic	24 (77.4%)
Black/African-American non-Hispanic	4 (12.9%)
Asian + white non-Hispanic	2 (6.5%)
Native Hawaiian + white non-Hispanic	1 (3.2%)
Age at follow-up (y)^	9.4y (2.1y)

*n (%), ^mean (standard deviation)

### Methylation stability over time

In the 24 children who had both cord blood and salivary samples available, global DNA methylation patterns between cord blood and saliva in childhood at the age of 6–12 years across 842,890 CpG sites (after excluding 3,613 CpG sites that are significantly different between blood and saliva [12]) remained stable (*R*^2^ range 0.891–0.965) (Table 2; Additional file 1: Table S1 with 95% CI). This remained true after limiting the analysis to 90,711 previously identified age-related CpGs from adult patients with colon cancer samples (Ghosh, *unpublished data)* (*R*^2^ 0.924–0.973), as well as across 14,238–53,403 age-related CpGs identified by Tajuddin et al. [13] as follows. Specifically looking across 53,403 age-related CpGs identified in African-Americans, the DNA methylation patterns of the African-American (AA) children from our cohort (Children F, Ja, Jb, and U) demonstrated high correlation over time (*R*^2^ range 0.871–0.959). Similarly, looking across 26,231 age-related CpGs identified in white (EA) individuals, the DNA methylation patterns for the 19 white children in our cohort were also high (*R*^2^ range 0.855–0.947). Considering only the 14,238 age-related CpGs that were in common between the African-American and white individuals in the Tajuddin et al. cohort, correlation in all 24 children remained high (*R*^2^ range 0.846–0.945).

**Table 2:** Correlation of DNA methylation between cord blood at birth and saliva in childhood

Family	Correlation (*R*^2^) using all CpGs*	Correlation using Ghosh 90 k age-related CpGs	Correlation using Tajuddin 53 k AA age-related CpGs	Correlation using Tajuddin 26 k EA age-related CpGs	Correlation using Tajuddin 14 k union {AA + EA} age-related CpGs
A	0.931	0.951	0.932	0.913	0.909
B˜	0.929	0.946	0.920	0.906	0.902
C˜	0.923	0.941	0.908	0.889	0.884
Da	0.941	0.960	0.935	0.914	0.911
Db	0.936	0.956	0.928	0.910	0.906
E	0.952	0.958	0.958	0.940	0.939
F^	0.891	0.926	0.871	0.855	0.846
G	0.965	0.973	0.959	0.947	0.945
I	0.922	0.943	0.909	0.894	0.888
Ja^	0.942	0.958	0.938	0.920	0.916
Jb^	0.920	0.941	0.911	0.894	0.885
Ka	0.926	0.946	0.925	0.902	0.900
Kb	0.924	0.940	0.923	0.903	0.899
M	0.927	0.951	0.915	0.897	0.891
N	0.955	0.970	0.953	0.938	0.936
Oa	0.921	0.944	0.912	0.893	0.885
Ob	0.922	0.945	0.905	0.886	0.878
P	0.943	0.961	0.941	0.922	0.918
R	0.898	0.933	0.879	0.858	0.849
S	0.915	0.935	0.915	0.896	0.893
T	0.936	0.953	0.927	0.905	0.901
U^	0.908	0.931	0.897	0.880	0.870
V	0.932	0.951	0.927	0.903	0.900
W	0.950	0.964	0.946	0.930	0.926

*Excluding 3,613 CpGs significantly different between blood and saliva [12]

^Children whose parents identify as Black/African-American

˜Unassisted conceptions

### Outlier methylation phenotype

**Fig. 1:**
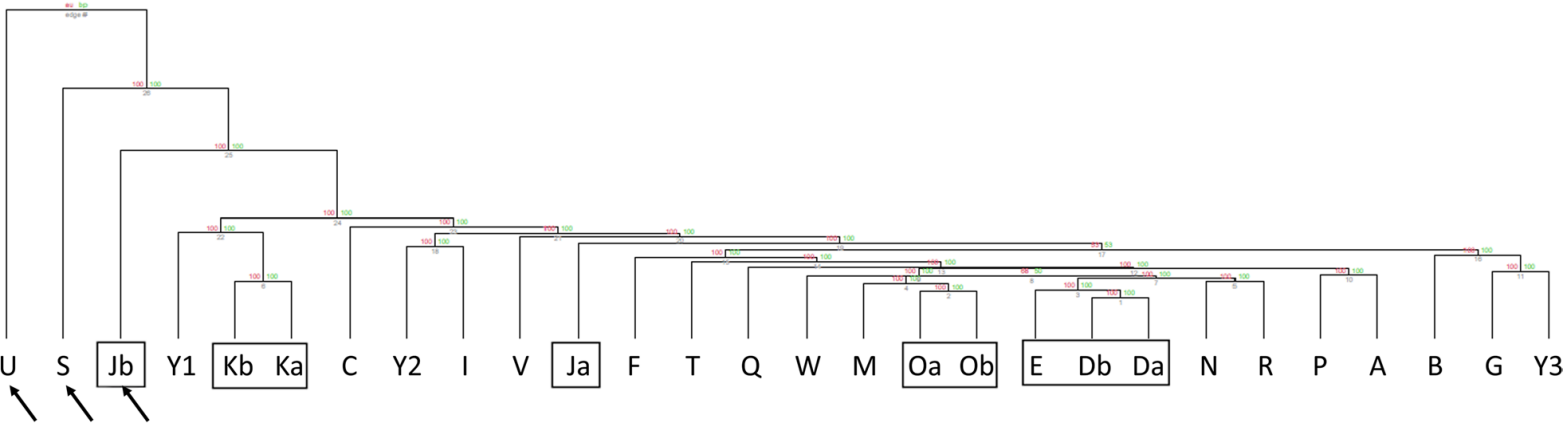
Hierarchical clustering analysis of cord blood CpGs after excluding XY sex-biased and SNP-linked (ancestry-based) CpG sites. The three OMP individuals (arrows) based on statistical calculations appear as outliers in this analysis as well. Families are designated with boxes. Individuals *Y*1, *Y*2, and *Y*3 are not related

At birth, three out of 28 children (Children Jb, S, and U) were identified to display an OMP based on statistical upper and lower limits in our cord blood dataset. The upper limit for the number of outlier CpGs in our dataset was identified to be 80,233; all three OMP individuals were significantly above that limit (189,873; 160,935; and 171,662, respectively). The OMP was confirmed for all three children when compared to upper and lower limits calculated from one external healthy cord blood dataset derived from 7 children (6 White, 1 Asian Indian, 1 Mixed-race) (GSE103189, [14]) and for two of three children (Children S and U) in another (GSE122288, unpublished data, race/ethnicity data not available). Furthermore, all three children appear to be outliers in hierarchical clustering analysis after excluding 74,747 XY-linked and SNP-linked CpGs (to prevent genetic sex and ancestry impacts on clustering) (Fig. 1). Of note, families clustered together except in the case of the one member of a dizygotic twin-pair who demonstrated an OMP (Child Jb).

A sensitivity analysis excluding the two children who were conceived without ART assistance yielded similar results, with the same three individuals (Jb, S, and U) confirmed to demonstrate an OMP (Additional file 2: Fig. S1).

**Fig. 2:**
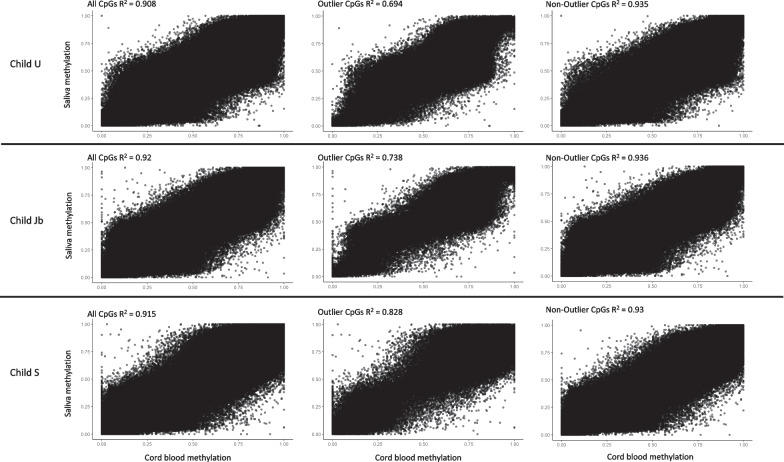
Correlation of DNA methylation between cord blood at birth and saliva in childhood in three children who demonstrate OMP at birth across all CpG sites, only their respective outlier CpG sites, and their non-outlier CpG sites

When analyzing only the outlier CpGs over time in each of the three OMP children, they remained stable overall (*R*^2^ values—U: 0.694, Jb: 0.738, and S: 0.828), although they showed greater variation than their respective non-outlier CpGs (*R*^2^ values—U: 0.935, Jb: 0.936, and S: 0.93) (Fig. 2).

### Ingenuity pathway analysis

Ingenuity pathway analysis (IPA) was performed on the common outlier CpGs in gene promoter regions in cord blood DNA from the three OMP children (Additional file 3: Table S2). IPA of the hypomethylated CpGs demonstrated enrichment for alterations in genes involved in the following canonical pathways: germ cell–Sertoli cell and Sertoli cell–Sertoli cell junction signaling, ephrin receptor signaling, and CXCR4 signaling. The gene *BCAR1,* which is important for cell migration in malignancies, was common to all four pathways. The gene *SORBS1* was hypomethylated in three of those four pathways; mutations in this gene are associated with insulin resistance.

IPA of the hypermethylated CpGs demonstrated enrichment in the following pathways: natural killer cell signaling, PI3K signaling in B lymphocytes, and IL4 signaling. Differentially methylated genes in these pathways included *PIK3CD*, *INPP5D*, and *VAV1*, all of which are involved in immune regulation and are associated with various malignancies.

Interestingly, IPA of the outlier CpG sites of one of the children demonstrated enrichment for alterations in genes critical for focal adhesion kinase (FAK) signaling, among others. The FAK signaling pathway, which plays a role in neural migration and synapse formation, has been associated with autism [15]. Notably, this child has been diagnosed with ADHD and her parents also reported concern for developmental delay and autism.

## Discussion

In this study, we have demonstrated that genome-wide methylation patterns remain stable between cord blood at birth and saliva in childhood at the age of 6–12 years. Furthermore, some individuals that are identified to display an OMP at birth continue to display it in childhood. This raises the possibility that certain childhood outcomes may be predictable based on DNA methylation analysis at birth.

Prior studies have identified persistent differences in DNA methylation over time after an environmental exposure. For example, prenatal exposure to maternal smoking can be identified in differentially methylated regions (DMRs) at birth, and these findings persist into childhood and adulthood [16–18]. In an analysis of twenty children with respiratory allergies compared to 20 control children using the Illumina Methylation 450 K BeadChip array, Boever et al. reported that there are specific DMRs in the saliva and blood of children that are relevant to respiratory allergies and further found that those methylation patterns were already present in the children’s bio-banked cord blood [19]. Specifically, they identified 83 DMRs in the blood of the 11-year-old children, 26 DMRs in their cord blood, and 5 DMRs in their saliva; two of these DMRs were in common between all three samples and were noted to be involved in pathways previously implicated in respiratory allergy disease.

Another study performed an epigenome-wide association study (EWAS) using the Illumina Methylation 450 K BeadChip array (and target validation using the Sequenom MassArray EpiTYPER) to compare methylation differences between twelve extreme preterm birth infants (born at < 31 weeks of gestational age) and twelve term infants at birth and 18 years of age [20]. Although the authors noted that the majority of significant methylation differences identified at birth were largely resolved by 18 years of age, they identified 10 probes (of 1555) that persisted to be significantly different over time, suggesting an ‘epigenetic legacy of preterm birth.’ Furthermore, they noted a significant overlap between CpGs that were differentially methylated at birth and those that changed by age 18 years. This is concordant with our study where we noted that the *R*^2^ values of the outlier CpGs over time were lower than those of CpGs that did not meet outlier criteria. This demonstration of increased variation over time is consistent with the hypothesis that these sites are likely more susceptible to environmental influences, including during the periconception period.

In contrast, other studies have not found that epigenetic differences identified at birth persist into childhood, suggesting some mechanism of self-correction. Specifically investigating the association of preterm birth with altered DNA methylation in genes encoding insulin-like growth factor 2 (IGF2) and FK506-binding protein 5 (FKBP5), Piyasena et al. [21] noted that the DMRs in these two genes were hypomethylated in the saliva of 50 preterm infants at birth compared to 40 term infants, but these differences were no longer identified at 1 year of age. Looking at the effects of maternal (i.e., prenatal) socioeconomic status on DNA methylation using the Illumina Methylation 450 K BeadChip in 609 children, Laubach et al. found that only one of 29 DMRs identified at birth in cord blood remained apparent in peripheral leukocytes in early childhood (age 3 years) and none persisted to mid-childhood (age 7 years) [22]. Similarly, another study exploring the effect of maternal mood disorders and the prescription of antidepressant medications during pregnancy found a single CpG site (using Illumina Methylation 450 K BeadChip) that was differentially methylated in two cohorts at birth (*n* = 479 and *n* = 999); in the smaller cohort, this difference persisted in early childhood (*n* = 120) but attenuated by mid-childhood (*n* = 460) [23].

Given these attenuations, it is prudent to evaluate whether this apparent loss of DNA methylation differences over time is simply due to age-related changes. Although one can calculate or predict an individual’s age based on epigenetic clocks derived from DNA methylation data, age-related DNA methylation changes are small. Prior studies have observed incremental, 0.1% per year, age-related DNA methylation changes at ~ 15–20% of CpG sites [24–28]. For example, in an EWAS of infant saliva between 6 and 52 weeks of age, Wilkenius et al. [29] demonstrated that only 42 genes in a total of 101 out of 423,315 probes demonstrated statistically significant DNA methylation changes over that time frame. This is consistent with our findings of a high correlation of DNA methylation over time across all CpG sites, age-related CpG sites (Table 1), and outlier CpG sites (Fig. 2).

This persistence of significant methylation differences in our study suggests that a methylome disrupted during the periconceptual period is like to continue to be disrupted in childhood, and potentially into adulthood. As we have previously reviewed [30], multiple studies have found that IVF-related procedures (e.g., superovulation and intra-cytoplasmic sperm injection) and manipulation of laboratory conditions (e.g., oxygen tension, temperature, humidity, and pH) can lead to alterations in DNA methylation in offspring. Furthermore, some, but not all prior studies, have identified DNA methylation differences between ART-conceived and non-ART-conceived children. We suspect that these inconsistent results may be due to both significant heterogeneity between the studies and the variable presence of individuals who are ‘epigenetic outliers’ similar to those identified in this study.

Of additional interest, we noted that families clustered together except in the case of the one member of a dizygotic twin-pair who demonstrated on OMP (Child Jb). As these were dizygotic twins, we expect them to behave more likely siblings than monozygotic twins, which suggests that there is something inherent to that individual that contributed to them being an outlier. Furthermore, an increasing number of studies have demonstrated that multiple factors, including genetic, environments, stochastic, and age/time, affect epigenetic variation and the resulting phenotypes, including in twins [31].

The major strength of this study is the prospective collection and longitudinal analysis of cord blood at birth and saliva in early childhood. With regards to identifying outliers, we used external cord blood datasets to confirm that individuals consistently demonstrated an OMP. We also reconfirmed the finding that DNA methylation patterns remain stable over time using externally identified age-related CpGs.

A potential limitation is that different tissues were used for analysis at birth and in childhood. It has been well characterized that ~ 15% of CpG sites exhibit tissue or cell-type-specific DNA methylation patterns. As such, differences in the cell composition of cord blood and saliva may contribute to differences in methylation patterns seen. Nevertheless, prior studies have shown that there is a high correlation in DNA methylation profiles between saliva and peripheral blood in adults and adolescents [12, 32, 33]. In the current study, deconvolution analyses using cell-type-specific differentially methylated regions to infer cell-type proportions (based on 8 CpG sites as described in [34]) demonstrated that the majority of the isolated cells from both saliva and blood were leukocytes (Additional file 4: Fig. S2). Despite this, we cannot completely exclude the possibility that ‘outlier’ patterns may be due to cell-composition differences.

Other limitations are as follows. First, the overall sample size remains small. The COVID-19 pandemic limited our ability to bring children from the cohort back in for follow-up visits in childhood. Future work will focus on the inclusion and analysis of more children from the original (and ongoing) large cohort.

Second, all but two of the children analyzed in this study were conceived via ART. Although we cannot comment from this study whether or not it was any specific ART exposure that led to the outlier phenotypes, our prior analyses have demonstrated that children conceived via ART are more likely to demonstrate an OMP than children conceived without assistance [11]. We included the unassisted conceptions in our analyses for several reasons; 1) to see if they demonstrate similar stability over time to the ART-conceived children (which they do), and 2) to maximize the number of children for whom paired samples were available. Furthermore, our sensitivity analyses identified that even after excluding the children not conceived via ART, the same three individuals demonstrate an OMP.

Third, while the majority of our study population had parents who identify as White Non-Hispanic, two out of the three children who demonstrated an OMP at birth had parents who identified as Black/African-American. We can thus not exclude that there may be racial/ethnic differences that contribute to DNA methylation differences. Furthermore, several children were unable to be included in the paired analyses due to lack of either their cord blood or their salivary sample. However, all available samples were used for calculating outlier status. Given the high correlations seen in all of the pair analyses, this is unlikely to have affected the results, and the additional data only strengthens the OMP status of the identified individuals. Finally, we lack a functional assessment of the differential methylation patterns on gene expression. Therefore, we cannot definitively conclude that the gene pathways identified are responsible for any differences in childhood diseases or other outcomes.

## Conclusions

DNA methylation signatures in cord blood remain stable over time as demonstrated by a strong correlation with epigenetic salivary signatures in childhood. More importantly, outlier methylation status persists and may be associated with undesirable clinical outcomes in childhood and beyond.

## Methods

### Study cohort

The study population includes a subset (*n* = 31) of a cohort of children after conception and/or delivery within the clinical practices of the University of Pennsylvania. Families undergoing ART at Penn Fertility Care as well as those who conceived without any medical assistance and who received their obstetrical care through the University of Pennsylvania System were enrolled in the original project. Information on demographic data, ART cycle parameters, and perinatal outcomes for all ART and unassisted pregnancies were collected via chart abstraction and from an existing clinical database. All data were input and managed using Research Electronic Data Capture (REDCap) which provides a secure, Web-based application, including audit trails [35, 36]. Cord blood DNA was collected and banked at the time of delivery. Long-term follow-up of these children was then performed at ages 6–12 years with either an at-home or outpatient clinic visit, at which time health history, cardiometabolic parameters, and salivary samples for DNA assessment were collected.

From the 31 children, we had 28 cord blood samples and 28 salivary samples. Of these, 24 could be included in paired analyses.

### DNA extraction

All procedures were performed according to the manufacturer’s instructions. Cord blood DNA was isolated using NucleoSpin Blood Columns (Macherey–Nagel). Cord blood DNA and salivary DNA were both extracted using PureLink Genomic DNA Mini Kit (Thermo Fisher Scientific).

### Methylation analysis

DNA samples were processed for Infinium MethylationEPIC BeadChip (Illumina) array after bisulfite conversion via EZ-DNA Methylation Kit (Zymo Research) using the standard cycling protocol. Both saliva and cord blood were run on the same array with randomized placement. IDAT files were processed into background-corrected data using the *minifi* Bioconductor R package after trimming probes with detection *p* values > 0.01 (~ 20,000). To evaluate global DNA methylation stability over time, we used Pearson’s correlation to calculate *R*^2^ values between cord blood DNA methylation and paired (i.e., same individual) saliva DNA methylation. We also confirmed our findings by looking only at age-related CpGs previously identified by Ghosh et al. and Tajuddin et al. [13]; these two cohorts were chosen because they uniquely had both white and black individuals included.

To identify OMP individuals, we then performed outlier calculations on beta values as described previously [37]*.* In brief, outlier CpGs were identified by having a beta value above or below the upper (quartile 3 + 1.5*IQR) and lower (quartile 1–1.5*IQR) limits, respectively, for that particular CpG site within our data set. OMP was then defined as individuals who have significantly more outlier CpGs than the other individuals within the cohort (number of outlier CpGs greater than the upper limit (quartile 3 + 1.5*IQR)). In this cohort, the upper limit was calculated to be 80,233 outlier CpGs, based on the 28 children from whom cord blood was available.

Unsupervised hierarchical clustering analysis was then done via the pvclust package in R, which uses a multiscale bootstrap resampling approach.

### Gene pathway analysis

A total of 20,834 CpGs were outliers in all three OMP individuals. Of these, 2384 were consistently hypermethylated and 13,448 were consistently hypomethylated. When narrowing only to promoter regions, 5534 CpGs were outliers in all three individuals with 902 hypermethylated and 3503 hypomethylated. We then identified the common 63 genes that were hypermethylated in their promoter regions (at least 3 CpGs within the promoter and 80% or more of the CpGs were hypermethylated) and 74 genes that were hypomethylated in their promoter regions (at least 5 CpGs within the promoter and 80% more of the CpGs were hypomethylated). These lists of genes were then uploaded into Ingenuity Pathway Analysis (QIAGEN) for core analysis. Core analysis was then performed with the following settings: human genes only with only experimentally observed direct relationships in all tissues and cell lines. Of note, there did not appear to be a correlation between the number of CpGs at the promoter and the list of hypermethylated or hypomethylated genes.

## Supplementary Information

**Additional file 1: Table S1.** Correlation of DNA methylation between cord blood at birth and saliva in childhood (95% CI).

**Additional file 2: Fig. S1.** Sensitivity hierarchical clustering analysis (including ART-conceived children only) of cord blood CpGs after excluding *XY* sex-biased and SNP-linked (ancestry-based) CpG sites. The same three individuals (arrows) as in the full analysis (Fig. 1) were identified to demonstrate an OMP.

**Additional file 3: Table S2.** The table shows the ingenuity canonical pathways, identified based on whether CpG sites were hypomethylated or hypermethylated, and the molecules/genes involved in each of those pathways. The *p*-value provided demonstrates the likelihood that the association between the genes and their identified pathways is due to random chance.

**Additional file 4: Fig. S2.** Deconvolution analysis, using cell-type specific differentially methylated regions to infer cell type proportions, demonstrates the proportion of cells that were leukocytes in each individual sample and how each of those tracks from cord blood to saliva.

## Data Availability

The datasets generated and analyzed during the current study are available from the corresponding author upon reasonable request.

## Abbreviations

ART: Assisted reproductive technologies
DMR: Differentially methylated region
IPA: Ingenuity pathway analysis
IVF: In vitro fertilization
OMP: Outlier methylation phenotype
