# Supplementary material for: Disrupted methylation patterns at birth persist in early childhood: a prospective cohort analysis
Source: Clin Epigenetics. 2022 Oct 15;14:129. doi: 10.1186/s13148-022-01348-x (PMC9568969; doi:10.1186/s13148-022-01348-x)
Supplement: Supplementary file 1 — Additional file 1: Table S1. Correlation of DNA methylation between cord blood at birth and saliva in childhood (95% CI). [file 13148_2022_1348_MOESM1_ESM.docx]

**Supplementary Table 1:** Correlation of DNA methylation between cord blood at birth and saliva in childhood (95% CI)

|  | All CpGs* | | Ghosh 90k  Age-Related CpGs | | Tajuddin 53k AA  Age-Related CpGs | | Tajuddin 26k EA  Age-Related CpGs | | Tajuddin 14k Union {AA+EA}  Age-Related CpGs | |
| --- | --- | --- | --- | --- | --- | --- | --- | --- | --- | --- |
| Family | R^2^ | 95% CI | R^2^ | 95% CI | R^2^ | 95% CI | R^2^ | 95% CI | R^2^ | 95% CI |
| A | 0.931 | (0.930,0.931) | 0.951 | (0.951,0.952) | 0.932 | (0.931,0.933) | 0.913 | (0.911,0.915) | 0.909 | (0.906,0.912) |
| B~ | 0.929 | (0.929,0.929) | 0.946 | (0.946,0.947) | 0.920 | (0.919,0.922) | 0.906 | (0.904,0.908) | 0.902 | (0.899,0.905) |
| C~ | 0.923 | (0.922,0.923) | 0.941 | (0.941,0.942) | 0.908 | (0.906,0.909) | 0.889 | (0.887,0.892) | 0.884 | (0.881,0.888) |
| Da | 0.941 | (0.941,0.942) | 0.960 | (0.959,0.961) | 0.935 | (0.934,0.936) | 0.914 | (0.912,0.916) | 0.911 | (0.908,0.914) |
| Db | 0.936 | (0.936,0.936) | 0.956 | (0.955,0.956) | 0.928 | (0.926,0.929) | 0.910 | (0.908,0.912) | 0.906 | (0.903,0.909) |
| E | 0.952 | (0.952,0.952) | 0.958 | (0.958,0.959) | 0.958 | (0.957,0.958) | 0.940 | (0.938,0.941) | 0.939 | (0.937,0.941) |
| F^ | 0.891 | (0.891,0.892) | 0.926 | (0.926,0.927) | 0.871 | (0.869,0.873) | 0.855 | (0.851,0.858) | 0.846 | (0.841,0.85) |
| G | 0.965 | (0.965,0.965) | 0.973 | (0.972,0.973) | 0.959 | (0.958,0.959) | 0.947 | (0.945,0.948) | 0.945 | (0.944,0.947) |
| I | 0.922 | (0.922,0.922) | 0.943 | (0.943,0.944) | 0.909 | (0.908,0.911) | 0.894 | (0.892,0.896) | 0.888 | (0.884,0.891) |
| Ja^ | 0.942 | (0.941,0.942) | 0.958 | (0.958,0.959) | 0.938 | (0.937,0.939) | 0.920 | (0.918,0.922) | 0.916 | (0.913,0.919) |
| Jb^ | 0.920 | (0.919,0.92) | 0.941 | (0.941,0.942) | 0.911 | (0.909,0.912) | 0.894 | (0.891,0.896) | 0.885 | (0.882,0.889) |
| Ka | 0.926 | (0.926,0.926) | 0.946 | (0.945,0.946) | 0.925 | (0.924,0.926) | 0.902 | ( 0.9,0.905) | 0.900 | (0.897,0.903) |
| Kb | 0.924 | (0.924,0.925) | 0.940 | (0.939,0.94) | 0.923 | (0.922,0.925) | 0.903 | ( 0.9,0.905) | 0.899 | (0.896,0.902) |
| M | 0.927 | (0.927,0.928) | 0.951 | (0.951,0.952) | 0.915 | (0.914,0.917) | 0.897 | (0.894,0.899) | 0.891 | (0.888,0.895) |
| N | 0.955 | (0.955,0.955) | 0.970 | (0.969,0.97) | 0.953 | (0.952,0.953) | 0.938 | (0.937,0.94) | 0.936 | (0.934,0.938) |
| Oa | 0.921 | (0.921,0.922) | 0.944 | (0.943,0.945) | 0.912 | (0.91,0.913) | 0.893 | (0.89,0.895) | 0.885 | (0.881,0.888) |
| Ob | 0.922 | (0.922,0.922) | 0.945 | (0.945,0.946) | 0.905 | (0.904,0.907) | 0.886 | (0.883,0.888) | 0.878 | (0.874,0.882) |
| P | 0.943 | (0.943,0.943) | 0.961 | (0.96,0.961) | 0.941 | (0.94,0.942) | 0.922 | (0.92,0.923) | 0.918 | (0.915,0.92) |
| R | 0.898 | (0.898,0.899) | 0.933 | (0.932,0.933) | 0.879 | (0.877,0.881) | 0.858 | (0.855,0.861) | 0.849 | (0.844,0.853) |
| S | 0.915 | (0.914,0.915) | 0.935 | (0.934,0.935) | 0.915 | (0.914,0.916) | 0.896 | (0.893,0.898) | 0.893 | (0.89,0.896) |
| T | 0.936 | (0.935,0.936) | 0.953 | (0.952,0.954) | 0.927 | (0.926,0.928) | 0.905 | (0.903,0.907) | 0.901 | (0.897,0.904) |
| U^ | 0.908 | (0.907,0.908) | 0.931 | (0.93,0.932) | 0.897 | (0.895,0.899) | 0.880 | (0.877,0.882) | 0.870 | (0.866,0.874) |
| V | 0.932 | (0.932,0.932) | 0.951 | (0.95,0.951) | 0.927 | (0.925,0.928) | 0.903 | (0.901,0.905) | 0.900 | (0.896,0.903) |
| W | 0.950 | (0.95,0.95) | 0.964 | (0.964,0.965) | 0.946 | (0.945,0.947) | 0.930 | (0.928,0.932) | 0.926 | (0.924,0.929) |

*Excluding 3,613 CpGs significantly different between blood and saliva (12)

^Children whose parents identify as Black/African American

˜Unassisted conceptions
