# Supplementary figures and images for: Disrupted methylation patterns at birth persist in early childhood: a prospective cohort analysis
Source: Clin Epigenetics. 2022 Oct 15;14:129. doi: 10.1186/s13148-022-01348-x (PMC9568969; doi:10.1186/s13148-022-01348-x)

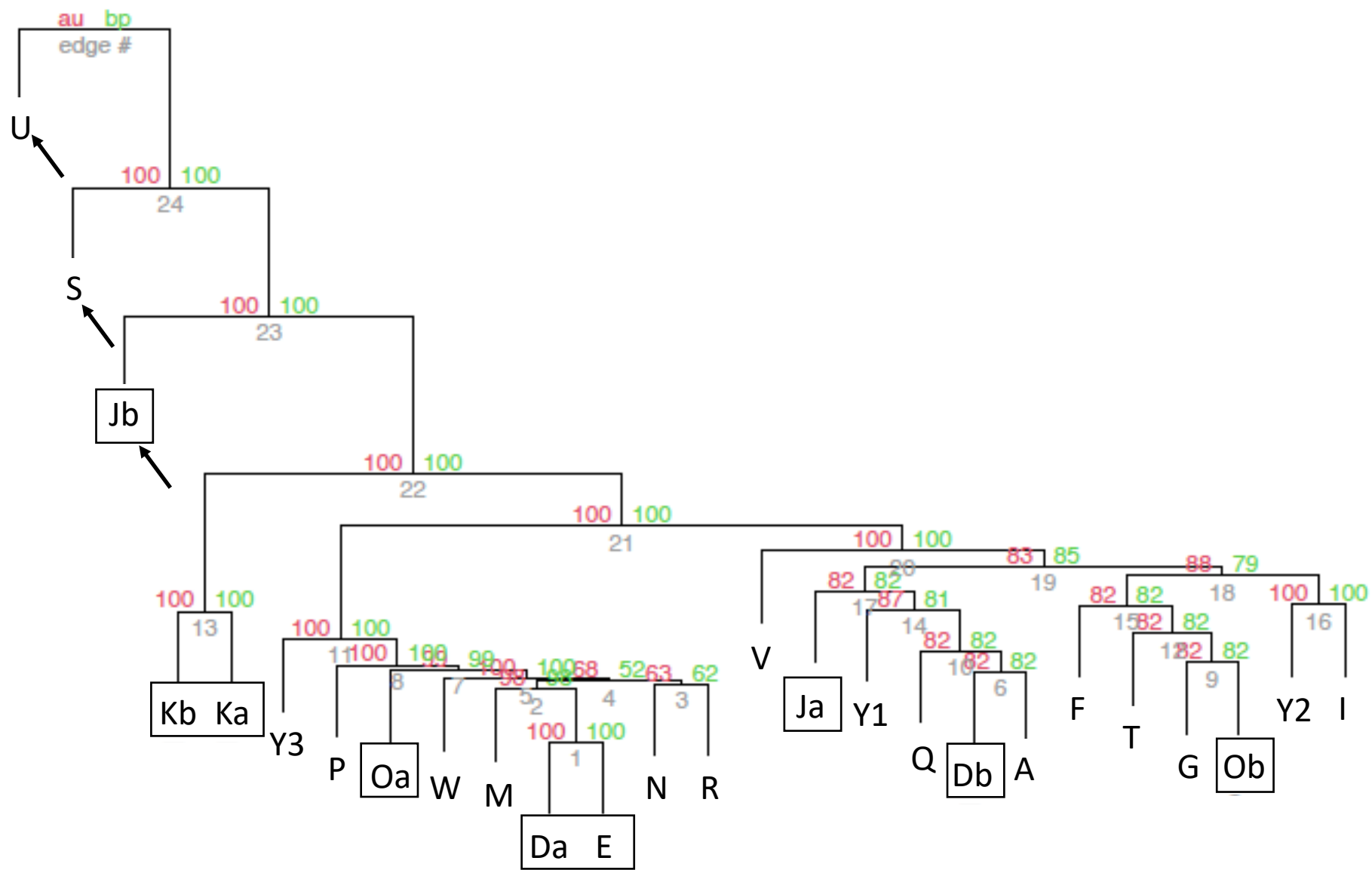

Supplement: Supplementary file 2 — Additional file 2: Fig. S1. Sensitivity hierarchical clustering analysis (including ART-conceived children only) of cord blood CpGs after excluding XY sex-biased and SNP-linked (ancestry-based) CpG sites. The same three individuals (arrows) as in the full analysis (Fig. 1) were identified to demonstrate an OMP. [file 13148_2022_1348_MOESM2_ESM.pdf]

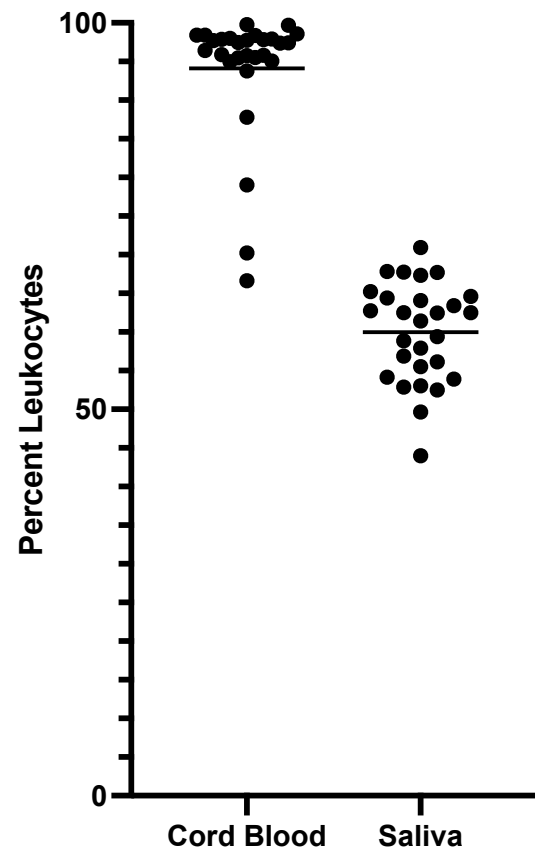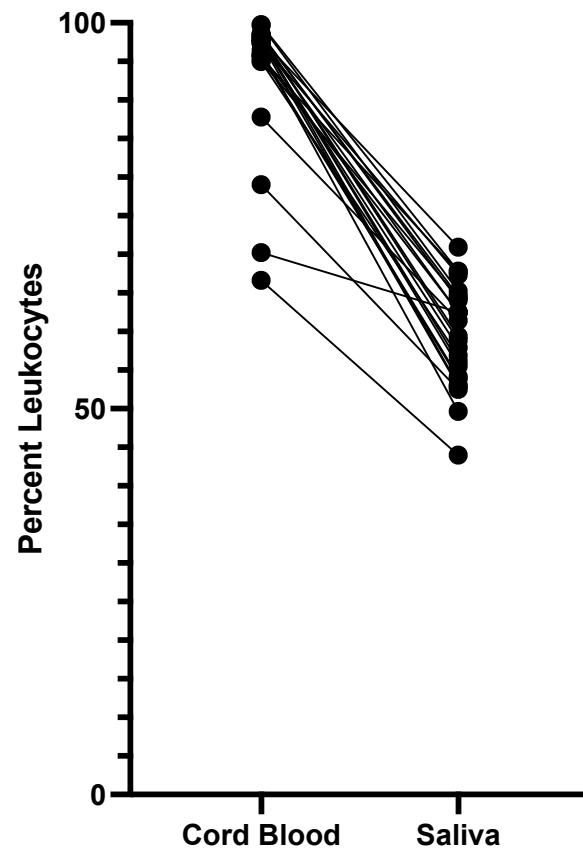

Supplement: Supplementary file 4 — Additional file 4: Fig. S2. Deconvolution analysis, using cell-type specific differentially methylated regions to infer cell type proportions, demonstrates the proportion of cells that were leukocytes in each individual sample and how each of those tracks from cord blood to saliva. [file 13148_2022_1348_MOESM4_ESM.pdf]
