# Supplementary material for: Disrupted methylation patterns at birth persist in early childhood: a prospective cohort analysis
Source: Clin Epigenetics. 2022 Oct 15;14:129. doi: 10.1186/s13148-022-01348-x (PMC9568969; doi:10.1186/s13148-022-01348-x)
Supplement: Supplementary file 3 — Additional file 3: Table S2. The table shows the ingenuity canonical pathways, identified based on whether CpG sites were hypomethylated or hypermethylated, and the molecules/genes involved in each of those pathways. The p-value provided demonstrates the likelihood that the association between the genes and their identified pathways is due to random chance. [file 13148_2022_1348_MOESM3_ESM.docx]

**Supplementary Table 2**

| **Hypomethylated CpGs** | | |
| --- | --- | --- |
| **Ingenuity Canonical Pathways** | ***p*-value (-log)** | **Molecules/Genes involved** |
| Germ Cell-Sertoli Cell Junction Signaling | 2.51 | BCAR1,JUP,PXN,SORBS1 |
| Ephrin Receptor Signaling | 2.21 | BCAR1,GNG7,PXN,SORBS1 |
| Sertoli Cell-Sertoli Cell Junction Signaling | 2.21 | BCAR1,CLDN4,JUP,SORBS1 |
| CXCR4 Signaling | 1.65 | BCAR1,GNG7,PXN |
| **Hypermethylated CpGs** | | |
| Natural Killer Cell Signaling | 4.68 | JAK3,LAIR1,LCP2,PIK3CD,PTPN6,VAV1 |
| PI3K Signaling in B Lymphocytes | 4.31 | CD79B,INPP5D,PIK3CD,PLCB2,VAV1 |
| Superpathway of Inositol Phosphate Compounds | 4.28 | INPP5D,PIK3CD,PLCB2,PPP1R16B,PTPN6,PTPN7 |
| IL-4 Signaling | 3.86 | INPP5D,JAK3,PIK3CD,PTPN6 |

The table shows the ingenuity canonical pathways, identified based on whether CpG sites were hypomethylated or hypermethylated, and the molecules/genes involved in each of those pathways. The *p*-value provided demonstrates the likelihood that the association between the genes and their identified pathways is due to random chance.
